# Cardiovascular risk factors indirectly affect acute post-stroke cognition through stroke severity and prior cognitive impairment: a moderated mediation analysis

**DOI:** 10.1186/s13195-020-00653-y

**Published:** 2020-07-16

**Authors:** Bogna A. Drozdowska, Emma Elliott, Martin Taylor-Rowan, Robert C. Shaw, Gillian Cuthbertson, Peter Langhorne, Terence J. Quinn

**Affiliations:** 1 Institute of Cardiovascular and Medical Sciences, University of Glasgow, Glasgow, UK; 2 School of Medicine, Dentistry & Nursing, University of Glasgow, Glasgow, UK

**Keywords:** Stroke, Cognition, Cardiovascular risk factors, Dementia, Mediation, Moderation

## Abstract

**Background:**

Cognitive impairment is an important consequence of stroke and transient ischaemic attack, but its determinants are not fully understood. Simple univariable or multivariable models have not shown clinical utility for predicting cognitive impairment. Cardiovascular risk factors may influence cognition through multiple, direct, and indirect pathways, including effects on prior cognition and stroke severity. Understanding these complex relationships may help clinical teams plan intervention and follow-up strategies.

**Methods:**

We analysed clinical and demographic data from consecutive patients admitted to an acute stroke ward. Cognitive assessment comprised Abbreviated Mental Test and mini-Montreal Cognitive Assessment. We constructed bias-corrected confidence intervals to test indirect effects of cardiovascular risk factors (hypertension, vascular disease, atrial fibrillation, diabetes mellitus, previous stroke) on cognitive function, mediated through stroke severity and history of dementia, and we assessed moderation effects due to comorbidity.

**Results:**

From 594 eligible patients, we included 587 in the final analysis (age range 26–100; 45% female). Our model explained *R*^2^ = 62.10% of variance in cognitive test scores. We found evidence for an indirect effect of previous stroke that was associated with increased risk of prevalent dementia and in turn predicted poorer cognitive score (estimate = − 0.39; 95% bias-corrected CI, − 0.75 to − 0.13; *p* = 0.02). Atrial fibrillation was associated with greater stroke severity and in turn with a poorer cognitive score (estimate = − 0.27; 95% bias-corrected CI, − 0.49 to − 0.05; *p* = 0.02). Conversely, previous TIA predicted decreased stroke severity and, through that, lesser cognitive impairment (estimate = 0.38; 95% bias-corrected CI, 0.08 to 0.75; *p* = 0.02). Through an association with reduced stroke severity, vascular disease was associated with lesser cognitive impairment, conditional on presence of hypertension and absence of diabetes mellitus (estimate = 0.36; 95% bias-corrected CI, 0.03 to 0.68; *p* = 0.02), although the modelled interaction effects did not reach statistical significance.

**Conclusions:**

We have shown that relationships between cardiovascular risk factors and cognition are complex and simple multivariable models may be overly reductionist. Including direct and indirect effects of risk factors, we constructed a model that explained a substantial proportion of variation in cognitive test scores. Models that include multiple paths of influence and interactions could be used to create dementia prognostic tools for use in other healthcare settings.

## Background

Cognitive impairment is an important yet under-researched complication of stroke and transient ischaemic attack (TIA). It has been recognised as both common, with estimates ranging up to even 96% [1], and highly relevant to survivors’ outcomes, predicting decreased quality of life, mood disorders, dependency, and mortality [2–4]. Features of the stroke or TIA only partly explain the variation seen in postmorbid cognitive function [5, 6]. Identifying other outcome determinants could offer mechanistic insights into cognitive decline, as well as improve risk stratification and intervention planning. Prognosis of cognitive function following stroke or TIA is, however, difficult. Although many multivariable prognostic models have been described, none are yet considered suitable for clinical use.

In this context, cardiovascular diseases are of particular interest, as they commonly co-occur in stroke populations and can potentially be modified. Cardiovascular risk factors are recognised as predictors of age-related cognitive decline and dementia [7, 8], with associations being reported for diabetes mellitus, hypertension, coronary and peripheral vascular disease, atrial fibrillation, and previous stroke [9–11]. Often these factors pre-date incident stroke and so it seems plausible that post-stroke cognitive impairment may be a manifestation of prevalent vascular neurodegenerative processes [5, 12]. However, the relationship of cardiovascular diseases to post-stroke cognition is likely not only to be driven by pre-stroke cognitive decline. Certain cardiovascular risk factors are associated with stroke severity, which is in turn a major determinant of cognitive outcome [13].

Intuitively, it seems that the presence of cardiovascular risk factors should be consistently detrimental across outcomes. For example, this seems to be the case for atrial fibrillation, which is associated with both higher incidence of dementia and greater stroke severity [14, 15]. However, the cognitive effects of other cardiovascular risk factors may be more complex, particularly where pathophysiological processes trigger endogenous adaptive mechanisms. For example, transient ischaemia can induce a state of ischaemic tolerance or preconditioning that temporarily (days to weeks) protects tissue from subsequent, persistent ischaemia [16]. Evidence from observational studies suggests that this phenomenon may occur in clinical practice. Indeed, stroke preceded by TIA has been associated with less severe symptoms, smaller infarct volumes, and better functional outcomes [17–19].

A similar example relates to collateral circulation. The presence of robust collaterals is also associated with favourable post-stroke clinical outcomes, including an improved response to thrombolytic and recanalisation therapy [20, 21]. Yet collateral development is, in part, driven by a pathological process, where chronic vascular disease leads to subclinical ischaemia [22–24]. To add further complexity, cardiovascular risk factors may not only co-occur but also interact, affecting the manifestation of these putative protective mechanisms. Findings from clinical and preclinical models suggest that both hypertension and diabetes may impair the development of collaterals [22, 25–29], while diabetes also prevents ischaemic tolerance [30, 31].

Although many studies have investigated the effect of cardiovascular risk factors on post-stroke cognition, results are inconclusive or conflicting [6, 13]. The traditional approach of using multivariable models can only identify those determinants directly associated with an outcome, while remaining relevant factors are held constant. Although in some cases this approach is sufficient, it does not allow us to explore the potential for multiple routes of predictor impact, nor the interaction between co-occurring diseases. It is possible that neutral results from previous research stem from the duality or conditionality of the studied effects.

We aimed to investigate how cardiovascular risk factors can affect cognitive function in the acute phase after stroke, through influence on stroke severity and prior cognitive impairment. We hypothesise that some effects may be conditional (on presence vs absence of diabetes/hypertension) and may differ depending on the path of influence, not only in strength, but also in direction. We assume the latter may occur for prior TIA and vascular disease, on the one hand associated with poorer cognitive performance through increased risk of prevalent dementia, while on the other potentially associated with improved performance through alleviated stroke severity.

## Methods

We conducted a secondary analysis of cross-sectional data, collected from patients admitted to an urban teaching hospital in the UK. The West of Scotland Research Ethics Committee (16/WS/0001) approved the primary project, allowing inclusion of participants without the requirement of providing written informed consent, data collection being embedded in routine clinical care. We based the design and conduct of the present study on recommendations from recent works, summarising theoretical and practical approaches to development of mediation and moderation models with an emphasis on best practice [32, 33]. In reporting our study, we followed Strengthening the Reporting of Observational Studies in Epidemiology (STROBE) guidelines [34].

### Study setting and participants

The primary study involved recruitment of consecutive patients admitted to a hyper-acute stroke unit. The unit provides high dependency clinical care, accepting all cases of suspected stroke and TIA, regardless of preadmission physical and cognitive function. Collection of anonymised data took place in four waves: May 2016 to February 2017, April to June 2017, October to December 2017, and July to August 2018. For the purpose of the present study, we only included those patients where the final consensus diagnosis by the clinical team was of stroke or TIA.

### Data collection

Five trained researchers used medical records and data collected by the clinical team during acute admission to extract information on basic demographics, pre-existing medical conditions, and findings from neurological examination. The researchers supplemented the clinical and demographic data with cognitive data based on their direct application of a standardised cognitive assessment for all study participants.

#### Predictors

We included cardiovascular risk factors which have been found to be associated with post-stroke cognitive function: vascular disease (peripheral and coronary), atrial fibrillation, hypertension, diabetes mellitus, previous stroke, and previous TIA. The clinical process in the stroke service is for these data to be confirmed from at least two data sources and includes information from both primary and secondary care records. We coded all risk factors as either present or absent. We also included information on basic demographics of sex and age, the latter treated as a continuous variable.

#### Mediators

In the primary study, stroke severity was assessed using the National Institutes of Health Stroke Scale (NIHSS) [35, 36]. Where a specific NIHSS score had not been documented by the clinical team, NIHSS score was derived based on findings from acute neurological examination described in patient notes [37]. As per emergency department triage policy, these examinations are performed immediately upon hospital admission and confirmed in the hyper-acute stroke unit, noting any changes in initial symptoms (resolution or progression). For inclusion in the analysis, we categorised NIHSS into four groups: no stroke signs (score of 0), minor stroke (score of 1 to 4), moderate stroke (score of 5 to 15), and severe stroke (score of 16 to 42) [38].

The second mediator we included in the model was a diagnosis of dementia prior to incident stroke or TIA. Dementia diagnosis was taken from primary or secondary care medical records, including records from mental health services. In the UK, dementia is diagnosed by secondary (specialist) care providers, based on the International Classification of Diseases (ICD) criteria [39].

#### Cognitive outcome

Cognitive performance was assessed within a week of stroke or TIA, using a short test battery of 13 items, comprising Hodkinson’s Abbreviated Mental Test Score (AMT-10) [40, 41] and a short form version of the Montreal Cognitive Assessment (MoCA) [42]. The specific tasks included in this measure have been described in a previous publication [43].

For the purpose of this study, we considered outcome data to be missing under three conditions: the patient refused to participate; the patient was discharged prior to assessment; the assessment was initiated but could not be completed due to external circumstances, e.g. to avoid disruption to activities carried out by the clinical team. If a patient was unable to complete a particular task due to an existing impairment (e.g. aphasia or limb weakness), we assigned a score of zero for that item, including it in the sum score [44]. In cases where the severity of the patient’s condition (e.g. drowsiness, agitation) prevented us from attempting the assessment altogether, we used the approach of other acute screening tests, which is to assign an untestable status the lowest score [45, 46]. Here, we assigned a score of − 1. This approach minimises missing data and avoids exclusion of patients with the most severe presentation. For analysis, we divided cognitive scores into quintiles, creating the following groups: (1) scores from − 1 to 2, (2) scores from 3 to 8, (3) scores from 9 to 11, (4) scores of 12 and 13, and (5) scores of 14 and 15.

### Statistical analysis

**Fig. 1:**
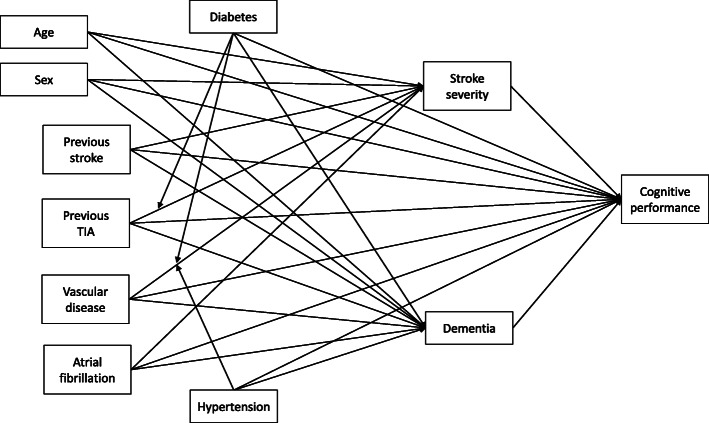
A conceptual diagram of the proposed dual moderated mediation model with two parallel mediators for acute cognitive performance

We developed a first stage dual moderated mediation model for prediction of cognitive performance, with two parallel mediators—stroke severity and previous diagnosis of dementia (Fig. 1). Although it was possible to model a number of different interaction terms between included predictors, we focused on three interactions most consistently demonstrated by existing evidence [22, 25–31]. Namely, we hypothesised that mediation of the effect of vascular disease and previous TIA on the outcome through stroke severity may be moderated by presence of diabetes (in both cases) and hypertension (for vascular disease only).

Following initial results, we removed from the final model the interaction term of TIA and diabetes mellitus that is shown in this figure (arrow pointing from diabetes to arrow between previous TIA and stroke severity).As mediation analyses assume causal relationships, we aimed to reflect the actual temporal order of occurrences in the model structure. This order was definite for paths mediated by stroke severity (with cardiovascular risk factors present before the index stroke/TIA, and the cognitive assessment carried out after) and seemed plausible for paths mediated by dementia, with evidence suggesting that cardiovascular diseases would have likely developed in preceding stages of life.

We analysed our data within a path analysis framework, using structural equation modelling software, MPLUS version 8.3 [47]. Having an ordinal outcome of interest, associations between variables were estimated based on probit regression, using a robust weighted least squares estimator (WLSMV). Missing data were handled as per software default; cases with missing data on predictors were removed from the analysis, while missing outcome data (here, including mediators) were treated as a function of the observed predictors [48].

Steps involved in the moderated mediation analysis included regressing cognitive performance on both mediators and the eight predictors, while each mediator was regressed on the eight predictors. In line with our hypothesis, we also regressed stroke severity on three interaction terms (TIA × diabetes mellitus, vascular disease × diabetes mellitus, vascular disease × hypertension). As the selection of variables for the analysis was based on research evidence, in order to avoid model overfitting, we intended to retain all predictors and both mediators, regardless of path significance. However, in order to achieve a more parsimonious model, we planned to remove nonsignificant interaction terms [49]. We used estimates obtained through the regression analyses to calculate indirect effects, applying a product of coefficients approach [33, 50].

For significant interaction terms, using the more complex example of vascular disease, we intended to firstly quantify the indices of partial mediated moderation; that is, how much the effect of vascular disease on cognitive performance through stroke severity changed depending on the following: firstly, the presence or absence of diabetes mellitus when hypertension is absent (held fixed, as all other predictors); secondly, the presence or absence of hypertension when diabetes mellitus is absent [51]. We then probed the partial moderated mediation effects to establish for what specific combination of factors (four options based on presence vs absence of diabetes and hypertension) vascular disease has a significant conditional indirect effect on cognitive performance. Based on the same principles, we planned to apply a simplified version of this procedure to estimate the conditional indirect effect of TIA depending on diabetes.

We determined the significance of individual paths and indirect effects through constructing bias-corrected bootstrap confidence intervals, based on drawing 1000 bootstrap samples. This method is recommended as it does not assume normal sampling distribution and offers greater precision for calculating confidence intervals (CIs) compared to alternatives [52, 53]. Previous bootstrapping studies have determined that in order to detect even small mediation and moderated mediation effects (estimate = 0.14) with 80% power, a sample of nearly 500 participants is required [54, 55].

To provide information on the magnitude of mediated effects, we calculated the proportion-mediated effect size, a ratio of the specific indirect effect to the total effect of a predictor [56]. This is considered an intuitive measure and is easily extrapolated from a simple to a multi-mediator model [32]. Expecting an inconsistent mediation model that is with direct and indirect effects of some predictors being of opposite signs, we planned to use absolute values of coefficients [57].

Recognising the potential bias from making assumptions about missing data, we repeated the described procedures in a sensitivity analysis, excluding participants who due to an existing impairment did not complete particular tasks for the cognitive assessment.

## Results

A total of 703 patients were admitted during study recruitment. From this sample, 109 were given a final diagnosis other than stroke or TIA, leaving 594 participants fulfilling inclusion criteria. Baseline characteristics are presented in Table 1. A correlation matrix for included variables is provided in Additional file 1 (Table S1). As seven patients had missing data on predictor variables, 587 participants were included in the final analysis.

**Table 1:** Baseline characteristics of study sample

Variables	
Age (years)	
Range	26 to 100
Median (IQR)	72.0 (21.0)
Missing	2
Sex (female)	
*N* (%)	269 (45.3%)
Missing	0
Previous stroke	
*N* (%)	136 (22.9%)
Missing	0
Previous TIA	
*N* (%)	40 (6.7%)
Missing	0
Hypertension	
*N* (%)	316 (53.2%)
Missing	5
Vascular disease	
*N* (%)	149 (25.1%)
Missing	5
Atrial fibrillation	
*N* (%)	108 (18.2%)
Missing	5
Diabetes	
*N* (%)	124 (20.9%)
Missing	5
Stroke severity (NIHSS score, range 0–42)	
Range for sample	0 to 31
Median (IQR)	3.0 (1–5)
Categories	
No stroke signs, *N* (%)	93 (16.1%)
Mild, *N* (%)	321 (55.4%)
Moderate, *N* (%)	128 (22.1%)
Severe, *N* (%)	37 (6.4%)
Missing	15
Prior dementia	
*N* (%)	57 (9.6%)
Missing	0
Cognitive test score (range 0–15)	
Range for testable participants	0 to 15
Median for testable participants (IQR)	11.0 (8–13)
Untestable patients, *N* (%)	101 (17.0%)
Missing	22

*TIA* transient ischaemic attack, *NIHSS* National Institutes of Health Stroke Scale

### Final model structure and properties

Our initial model included three interaction terms. However, preliminary results indicated that the interaction term of TIA and diabetes mellitus was not significantly associated with stroke severity (*p* = 0.560) and so we removed it from the model. As subsequent findings suggested a trend for the remaining two interaction terms, between vascular disease and diabetes mellitus (*p* = 0.057) and vascular disease and hypertension (*p* = 0.056), we opted to retain them. Therefore, the final model differed from that presented in Fig. 1 in only one aspect, namely, we did not consider diabetes as a moderator for the effect of TIA on stroke severity.

For this model, the chi-square statistic indicated no significant discrepancy between the observed and model-estimated covariance matrices: *χ*^2^ = 6.580, *p* = 0.254. Additional recommended fit indices confirmed good model fit: Root Mean Square Error of Approximation (RMSEA) = 0.023, Comparative Fit Index (CFI) = 0.995, and Standardised Root Mean Square Residual (SRMR) = 0.030 [58]. Overall, our model explained *R*^2^ = 62.10% of variance in cognitive test scores.

### Associations between predictors and mediators

More severe strokes were associated with age and atrial fibrillation, while severity decreased with a history of previous TIA. The observed associations with interaction terms, just above the threshold of statistical significance, suggested opposing effects of vascular disease, dependent on the presence of diabetes mellitus and hypertension. Co-occurring with the former, it appeared potentially associated with greater stroke severity, while co-occurring with the latter, it appeared associated with less severe presentation. Predictors of prior dementia included age and previous stroke. We also observed a trend for an association between dementia and history of vascular disease (*p* = 0.054).

### Direct effects on cognitive performance

We found that both mediators were associated with acute cognitive function: coefficient = − 0.748; 95% bias-corrected CI, − 0.963 to − 0.572 for stroke severity; coefficient = − 0.720; 95% bias-corrected CI, − 1.096 to − 0.444 for dementia. However, we observed no significant direct effects of included predictors on cognitive performance (Table 2).

**Table 2:** Direct associations between predictors and stroke severity, dementia, and cognitive performance

	Stroke severity (NIHSS)		Prior dementia		Cognitive performance	
	Unstandardised coefficient	95% bias-corrected CI	Unstandardised coefficient	95% bias-corrected CI	Unstandardised coefficient	95% bias-corrected CI
Age	0.012*	0.004–0.019	0.059*	0.039–0.078	0.003	− 0.016–0.027
Sex (female)	− 0.031	− 0.210–0.180	− 0.098	− 0.458–0.248	− 0.264	− 0.588–0.046
Previous stroke	0.008	− 0.217–0.222	0.538*	0.176–0.932	0.230	− 0.129–0.649
Previous TIA	− 0.512*	− 0.934 to − 0.147	− 0.342	− 3.886–0.279	− 0.141	− 2.136–0.510
Atrial fibrillation	0.355*	0.075–0.609	0.145	− 0.270–0.554	− 0.092	− 0.464–0.279
Diabetes	− 0.025	− 0.274–0.209	− 0.028	− 0.636–0.535	− 0.041	− 0.604–0.508
Hypertension	0.076	− 0.146–0.301	− 0.133	− 0.571–0.377	− 0.065	− 0.502–0.364
Vascular disease	0.002	− 0.405–0.374	0.611	− 0.051–1.246	0.390	− 0.178–1.127
Vascular disease × diabetes	0.466	− 0.031–0.924	–	–	–	–
Vascular disease × hypertension	− 0.486	− 0.971–0.016	–	–	–	–

*TIA* transient ischaemic attack, *NIHSS* National Institutes of Health Stroke Scale

*Significant at *p* < 0.05

### Indirect effects on cognitive performance

#### Effects mediated through stroke severity

We found that age had a negative specific indirect effect on cognitive performance (Table 3), with 16.36% of the absolute overall effect of age on cognition mediated by stroke severity. Poorer cognitive outcome was also indirectly associated with a history of atrial fibrillation, with a proportion-mediated effect size of 57.45%. Conversely, we observed improved cognitive performance through a specific indirect effect of previous TIA, which constituted 49.68% of the absolute overall effect.

**Table 3:** Indirect associations between predictors and cognitive performance

	Effects mediated through stroke severity (NIHSS)		Effects mediated through prior dementia	
	Unstandardised coefficient	95% bias-corrected CI	Unstandardised coefficient	95% bias-corrected CI
Age	− 0.009*	− 0.015 to − 0.003	− 0.043*	− 0.069 to − 0.024
Sex (female)	0.023	− 0.145–0.172	0.071	− 0.186–0.349
Previous stroke	− 0.006	− 0.175–0.168	− 0.387*	− 0.753 to − 0.130
Previous TIA	0.383*	0.083–0.745	0.247	− 0.218–2.152
Atrial fibrillation	− 0.266*	− 0.493 to − 0.052	− 0.105	− 0.479–0.202
Diabetes	0.019	− 0.161–0.207	0.020	− 0.398–0.516
Hypertension	− 0.057	− 0.244–0.109	0.096	− 0.278–0.476
Vascular disease	− 0.002	− 0.297–0.322	− 0.440	− 0.981–0.064
Vascular disease × diabetes	− 0.349	− 0.748–0.030	–	–
Vascular disease × hypertension	0.363	− 0.032–0.759	–	–

*TIA* transient ischaemic attack, *NIHSS* National Institutes of Health Stroke Scale

*Significant at *p* < 0.05

The indices of partial mediated moderation suggested a trend for both estimated conditional indirect effects of vascular disease on cognition through stroke severity (*p* = 0.077 for both diabetes mellitus and hypertension). Through probing, we observed that vascular disease produced a significant positive effect on performance under only one condition, where there was a history of hypertension without diabetes mellitus (estimate = 0.362; 95% bias-corrected CI, 0.032 to 0.675; *p* = 0.024). The proportion-mediated effect size was 30.37%.

#### Effects mediated through prior dementia

In relation to our second mediator, we found that previous stroke had a negative specific indirect effect on cognition, constituting 62.12% of the absolute overall effect. Despite the observed trend for an association between dementia and vascular disease, the specific indirect effect of this risk factor on cognitive performance did not reach statistical significance (*p* = 0.089). Age, therefore, was the only predictor to exert a significant negative indirect effect on cognition through both mediators. Compared to stroke severity, dementia conveyed a considerably larger portion of its overall absolute effect—78.18%.

### Sensitivity analysis

For the sensitivity analysis, we excluded 38 participants who due to impairments were not able to complete particular cognitive tasks. Estimates of direct and indirect effects are presented in Additional file 1 (Tables S2 and S3). Overall, the findings were similar to those presented for the main analysis, with differences specifically relating to associations with dementia. Namely, we noted a reversed situation for dementia predictors, where here the association with vascular disease was statistically significant and with previous stroke—at trend level (*p* = 0.056). Moreover, the indirect effect of previous stroke on cognition through dementia did not reach statistical significance (*p* = 0.080).

## Discussion

Based on data from a real-world sample of stroke unit inpatients, the results of our study supported the mediatory role of stroke severity and prior cognitive impairment in the effects of specific cardiovascular risk factors on acute cognition. Our analyses serve two purposes. By using a complex approach to capture direct, indirect, and interaction effects, we have created a model that reasonably predicts cognition following stroke or TIA, and may offer improvements on previous reductionist multivariable analyses. Accurate prognostic tools could improve clinical services, in particular allowing for better informed intervention planning and follow-up. At a methodological level, we have shown that attempts to understand the relationship between cardiovascular risk factors and cognition need to account for various life course exposures that may interact in unexpected ways.

Some of our findings are in line with previously reported associations and seem intuitively correct. Poorer cognitive performance was associated with the following: atrial fibrillation through increased stroke severity, previous stroke through increased risk of prevalent dementia, and with age through both mediators. Importantly, however, our results also suggest novel associations. For example, history of TIA and vascular disease—considered risk factors for cognitive impairment—may in some cases be associated with better acute cognitive performance through alleviating stroke severity.

Not all findings were consistent with our hypotheses. Perhaps most interestingly, we observed that the likely positive effect of vascular disease on cognition was conditional on the simultaneous absence of diabetes and history of hypertension. We assumed that the latter would be detrimental, with previous studies showing that high acute blood pressure, a state often seen in patients with chronic hypertension [59], is associated with poorer prognosis after stroke [60]. It seems, therefore, that the relationship between hypertension and post-stroke outcomes may be indeed more complex than previously suggested. The authors of a recent clinical study found that in cases of major stroke reperfusion, acute high blood pressure was associated with better collateral flow and thus decreased infarct growth and better clinical outcomes, while the opposite was observed for patients without reperfusion [61].

It is also important to note that the indices of partial mediated moderation did not reach statistical significance, and so we cannot conclude that there is a difference in the effect of vascular disease between patients with and without diabetes and hypertension. Moreover, we did not have access to data relevant to explaining mechanisms underlying analysed associations, for example, on degree of carotid stenosis and extent of collateral development, or time elapsed between previous TIA and subsequent stroke or TIA.

Although our hypotheses were formulated in view of potential endogenous adaptive processes, there are plausible alternative explanations. Treatment effects, which we were not able to control for, seem of particular importance here. Specifically, research findings suggest that aspirin, routinely administered following TIA, reduces the severity of early subsequent stroke [62], while statins, prescribed in cases of vascular disease, enhance collateral circulation [63, 64]. We were moreover unable to adjust for the effects of education, not only relevant to cognitive performance, but also associated with cardiovascular risk factor prevalence and outcomes [65]. These and other factors could be used to enhance future models. We do not claim that our model is definitive, but it allows to highlight the complex and sometimes counterintuitive relationships between cardiovascular risk factors and cognition.

Further study limitations relate to a retrospective assessment of risk factors from medical case records, as it is possible that relevant conditions had not been mentioned in notes or even diagnosed. We were also unable to verify whether participants experienced milder forms of cognitive impairment prior to incident stroke. Dichotomising into those cognitively intact and with a severe form of impairment does not reflect the true, gradual nature of cognitive deterioration. Finally, we did not have access to information on longer-term cognitive outcomes. Longitudinal studies have demonstrated considerable individual changes in cognitive status between the acute and chronic stages after stroke [4, 66, 67]. Nonetheless, early post-stroke cognitive impairment has been shown to be an important predictor of future patient outcomes, both cognitive and functional [2], and in healthcare settings, for many stroke survivors, the only opportunity to undergo a cognitive screen may be during hospital admission.

Strengths of our study include involving a sample representative of a real-world stroke population, while avoiding bias due to exclusion of patients with the most severe impairments. At the same time, we conducted a sensitivity analysis in a subgroup of participants with complete cognitive data to reflect a more conservative approach. We also strove to adhere to current best practice guidelines for design and conducting of mediation and moderated mediation analyses.

Although we consider our results as preliminary, they are not without clinical implications. Currently, there is a lack of consensus guidelines on delivering assessments and care focused on cognition following stroke or TIA [68, 69]. Most stroke services have insufficient resources to address these issues consistently for all patients, and therefore, it is important to identify who is most likely to experience cognitive impairment. Our study highlights the need to account for comorbidity and the potential for risk factors not only to co-occur, but also to interact. Although further confirmation is necessary, it seems plausible that for patients with a history of TIA and vascular disease with hypertension, the risk of cognitive impairment could be underestimated, as they are more likely to present with less severe strokes, while still being prone to the progressive neurodegenerative effects of these conditions, demonstrated in previous studies.

Taking into account that effects may differ in direction depending on the path of influence is also an important consideration for future studies, aiming to improve outcome prognosis and investigate the detrimental role of comorbidity, or the benefits of endogenous adaptive mechanisms and disease management. We may also gain a better understanding of associations by capturing milder forms of cognitive decline. Finally, it seems important to explore how the role of comorbidity in shaping cognitive outcomes may change over time, particularly as the length of life following stroke is increasing [70].

## Conclusions

Our findings highlight the importance and complex nature of relationships between cardiovascular diseases and post-stroke cognition. They further add to the body of evidence indicating the necessity to account for comorbidity when attempting to assess and understand cognitive changes following stroke, in both clinical and research contexts. In order to develop more precise prognostic models of post-stroke cognitive outcome, future studies should consider the potential for multiple paths of influence and interactions between predictors.

## Supplementary information

**Additional file 1: Table S1.** Correlations between variables included in the model for prediction of acute post-stroke cognitive performance. **Table S2.** Direct associations between predictors and stroke severity, dementia and cognitive performance. **Table S3.** Indirect associations between predictors and cognitive performance.

## Data Availability

The datasets analysed during the current study are now archived and available in the NHS Greater Glasgow and Clyde Safe Haven: https://www.nhsggc.org.uk/about-us/professional-support-sites/safe-haven/. Applications to access these data or other related data can be discussed with the Safe Haven Manager: safehaven@ggc.scot.nhs.uk.

## Abbreviations

AMT-10: Hodkinson’s Abbreviated Mental Test Score
CFI: Comparative Fit Index
CI: Confidence interval
MoCA: Montreal Cognitive Assessment
NIHSS: National Institutes of Health Stroke Scale
RMSEA: Root Mean Square Error of Approximation
SRMR: Standardised Root Mean Square Residual
STROBE: Strengthening the Reporting of Observational Studies in Epidemiology
TIA: Transient ischaemic attack
WLSMV: Robust weighted least squares estimator
