# Supplementary material for: Cardiovascular risk factors indirectly affect acute post-stroke cognition through stroke severity and prior cognitive impairment: a moderated mediation analysis
Source: Alzheimers Res Ther. 2020 Jul 16;12:85. doi: 10.1186/s13195-020-00653-y (PMC7367370; doi:10.1186/s13195-020-00653-y)
Supplement: Supplementary file 1 — Additional file 1: Table S1. Correlations between variables included in the model for prediction of acute post-stroke cognitive performance. Table S2. Direct associations between predictors and stroke severity, dementia and cognitive performance. Table S3. Indirect associations between predictors and cognitive performance. [file 13195_2020_653_MOESM1_ESM.docx]

**Additional file 1**

Table S1. Correlations between variables included in the model for prediction of acute post-stroke cognitive performance.

|  | Age | Sex | Previous stroke | Previous TIA | Atrial fibrillation | Diabetes | Hypertension | Vascular disease | Stroke severity | Dementia | Cognitive performance |
| --- | --- | --- | --- | --- | --- | --- | --- | --- | --- | --- | --- |
| Age |  | 0.276* | 0.044 | 0.159* | 0.453* | 0.047 | 0.261* | 0.233* | 0.175* | 0.575* | -0.425* |
| Sex (female) | 0.276* |  | -0.036 | 0.093 | -0.032 | -0.018 | 0.137* | 0.060 | 0.028 | 0.104 | -0.179* |
| Previous stroke | 0.044 | -0.036 |  | -0.404* | 0.087 | 0.294* | 0.192* | 0.197* | 0.039 | 0.260* | -0.088 |
| Previous TIA | 0.159* | 0.093 | -0.404* |  | -0.068 | -0.019 | 0.018 | 0.147 | -0.237* | -0.068 | 0.104 |
| Atrial fibrillation | 0.453* | -0.032 | 0.087 | -0.068 |  | 0.132 | 0.228* | 0.110 | 0.243* | 0.246* | -0.309* |
| Diabetes | 0.047 | -0.018 | 0.294* | -0.019 | 0.132 |  | 0.290* | 0.374* | 0.079 | 0.066 | -0.087 |
| Hypertension | 0.261* | 0.137* | 0.192* | 0.018 | 0.228* | 0.290* |  | 0.295* | 0.060 | 0.035 | -0.087 |
| Vascular disease | 0.233* | 0.060 | 0.197* | 0.147 | 0.110 | 0.374* | 0.295* |  | -0.031 | 0.291* | -0.021 |
| Stroke severity | 0.175* | 0.028 | 0.039 | -0.237* | 0.243* | 0.079 | 0.060 | -0.031 |  | 0.246* | -0.550* |
| Dementia | 0.575* | 0.104 | 0.260* | -0.068 | 0.246* | 0.066 | 0.035 | 0.291* | 0.246* |  | -0.609* |
| Cognitive performance | -0.425* | -0.179* | -0.088 | 0.104 | -0.309* | -0.087 | -0.087 | -0.021 | -0.550* | -0.609 |  |

*significant at p < 0.05

TIA, transient ischaemic attack

Stroke severity was assessed using the National Institutes of Health Stroke Scale (NIHSS).

Coefficients were obtained from correlations appropriate to each pair of variables: tetrachoric for two dichotomous, biserial for one dichotomous and one continuous, polychoric for two ordered categorical and one ordered categorical and one dichotomous, polyserial for one ordered categorical and one continuous.

**Results of sensitivity analysis**

Both mediators were associated with acute cognitive function: coefficient= -0.723; 95% bias-corrected CI: -0.943 to -0.550 for stroke severity; and coefficient = -0.670; 95% bias-corrected CI: -1.114 to -0.397 for dementia.

Table S2. Direct associations between predictors and stroke severity, dementia and cognitive performance.

|  | **Stroke severity (NIHSS)** | | **Dementia** | | **Cognitive performance** | |
| --- | --- | --- | --- | --- | --- | --- |
|  | Unstandardised coefficient | 95% bias-corrected CI | Unstandardised coefficient | 95% bias-corrected CI | Unstandardised coefficient | 95% bias-corrected CI |
| Age | 0.012* | 0.004 – 0.020 | 0.061* | 0.037 – 0.081 | 0.002 | -0.017 – 0.025 |
| Sex (female) | -0.066 | -0.254 – 0.141 | -0.080 | -0.444 – 0.296 | -0.188 | -0.526 – 0.157 |
| Previous stroke | 0.033 | -0.184 – 0.259 | 0.396 | -0.053 – 0.773 | 0.103 | -0.278 – 0.517 |
| Previous TIA | -0.394* | -0.776 – 0.006 | -0.256 | -1.163 – 0.305 | -0.012 | -0.727 – 0.571 |
| Atrial fibrillation | 0.373* | 0.072 – 0.641 | 0.053 | -0.427 – 0.497 | -0.124 | -0.508 – 0.351 |
| Diabetes | -0.035 | -0.307 – 0.246 | 0.009 | -0.771 – 0.556 | -0.022 | -0.671 – 0.522 |
| Hypertension | 0.116 | -0.161 – 0.328 | -0.234 | -0.762 – 0.270 | -0.081 | -0.554 – 0.318 |
| Vascular disease | 0.047 | -0.382 – 0.450 | 0.644* | -0.016 – 1.265 | 0.339 | -0.246 – 1.066 |
| Vascular disease X diabetes | 0.456 | -0.024 – 0.956 |  |  |  |  |
| Vascular disease X hypertension | -0.482 | -0.983 – 0.006 |  |  |  |  |

*significant at p < 0.05

TIA, transient ischaemic attack; NIHSS, National Institutes of Health Stroke Scale

Table S3. Indirect associations between predictors and cognitive performance.

|  | **Effects mediated through stroke severity (NIHSS)** | | **Effects mediated through dementia** | |
| --- | --- | --- | --- | --- |
|  | Unstandardised coefficient | 95% bias-corrected CI | Unstandardised coefficient | 95% bias-corrected CI |
| Age | -0.008* | -0.015 – -0.003 | -0.041* | -0.069 – -0.021 |
| Sex (female) | 0.048 | -0.104 – 0.197 | 0.054 | -0.214 – 0.307 |
| Previous stroke | -0.024 | -0.195 – 0.138 | -0.265 | -0.599 – 0.001 |
| Previous TIA | 0.285 | -0.020 – 0.584 | 0.171 | -0.217 – 0.826 |
| Atrial fibrillation | -0.269* | -0.492 – -0.049 | -0.035 | -0.387 – 0.270 |
| Diabetes | 0.025 | -0.195 – 0.234 | -0.006 | -0.393 – 0.542 |
| Hypertension | -0.084 | -0.263 – 0.110 | 0.157 | -0.165 – 0.588 |
| Vascular disease | -0.034 | -0.348 – 0.290 | -0.432 | -1.017 – -0.023 |
| Vascular disease X diabetes | -0.330 | -0.741 – 0.022 |  |  |
| Vascular disease X hypertension | 0.348 | -0.011 – 0.748 |  |  |

*significant at p < 0.05

TIA, transient ischaemic attack; NIHSS, National Institutes of Health Stroke Scale
